# Next-generation sequencing in familial breast cancer patients from Lebanon

**DOI:** 10.1186/s12920-017-0244-7

**Published:** 2017-02-15

**Authors:** Nadine Jalkh, Eliane Chouery, Zahraa Haidar, Christina Khater, David Atallah, Hamad Ali, Makia J. Marafie, Mohamed R. Al-Mulla, Fahd Al-Mulla, Andre Megarbane

**Affiliations:** 1 Unité de Génétique Médicale, Pôle Technologie Santé, Faculty of Medicine, Saint Joseph University, Beirut, Lebanon; 2 Trad Hospital, Beirut, Lebanon; 3 Department of Gynecology and Obstetrics, Hôtel-Dieu de France University Hospital, Saint Joseph University, Beirut, Lebanon; 4 Department of Medical Laboratory Sciences (MLS), Faculty of Allied Health Sciences, Health Sciences Center (HSC), Kuwait University, Safat, Kuwait; 5 Dasman Diabetes Institute (DDI), P.O Box 1180, Dasman, 15462 Kuwait; 6 Kuwait Medical Genetics Center, Maternity Hospital, Safat, Kuwait; 7 Department of Computing Sciences and Engineering, Kuwait University, P.O. Box 5969, Safat, 13060 Kuwait; 8 Health Sciences Center, Faculty of Medicine, Department of Pathology, Kuwait University, P.O.Box 24923, Safat, 13110 Kuwait; 9 Institut Jerome Lejeune, Paris, France

**Keywords:** Breast cancer, BRCA, Next-generation sequencing, Exome, Familial, Lebanon, Germline, Mutation

## Abstract

**Background:**

Familial breast cancer (BC) represents 5 to 10% of all BC cases. Mutations in two high susceptibility *BRCA1* and *BRCA2* genes explain 16–40% of familial BC, while other high, moderate and low susceptibility genes explain up to 20% more of BC families. The Lebanese reported prevalence of *BRCA1* and *BRCA2* deleterious mutations (5.6% and 12.5%) were lower than those reported in the literature.

**Methods:**

In the presented study, 45 Lebanese patients with a reported family history of BC were tested using Whole Exome Sequencing (WES) technique followed by Sanger sequencing validation.

**Results:**

Nineteen pathogenic mutations were identified in this study. These 19 mutations were found in 13 different genes such as: *ABCC12*, *APC*, *ATM*, *BRCA1*, *BRCA2*, *CDH1, ERCC6*, *MSH2*, *POLH*, *PRF1, SLX4*, *STK11* and *TP53*.

**Conclusions:**

In this first application of WES on BC in Lebanon, we detected six *BRCA1* and *BRCA2* deleterious mutations in seven patients, with a total prevalence of 15.5%, a figure that is lower than those reported in the Western literature. The p.C44F mutation in the *BRCA1* gene appeared twice in this study, suggesting a founder effect. Importantly, the overall mutation prevalence was equal to 40%, justifying the urgent need to deploy WES for the identification of genetic variants responsible for familial BC in the Lebanese population.

**Electronic supplementary material:**

The online version of this article (doi:10.1186/s12920-017-0244-7) contains supplementary material, which is available to authorized users.

## Background

Breast cancer (BC) is the most common cancer in women, accounting for around 25% of all new cases of cancer [1]. Most BC cases are sporadic, while 5 to 10% of all BC cases are inherited and cluster in families [1]. While mutations in *BRCA1* and *BRCA2* genes explain 16–40% of all familial BC cases [1–3], other genes have also been found to increase BC susceptibility, which highlights the polygenic nature of many BC cases [4]. Some of these genes including *CDH1*, *TP53*, *PTEN* and *STK11,* although less frequently altered compared to the *BRCA1/2* genes, they have been linked to high-penetrance autosomal dominant BC [5–7]. Moderate penetrance genes are implicated in around 5% of familial BC. These genes include the Fanconi anemia pathway genes: *FANCA*, *PALB2*, *BRIP1*, *RAD51C* and *XRCC2* [8–10] and non-Fanconi anemia genes: *ATM*, *CHEK2*, *NBN*, *RAD50*, *RAD51B*, and *RAD51D* [11–15].

In Lebanon, BC is the most common cancer type in females and it constitutes one-third of all reported cancer cases. BC incidence rates are expected to reach 137 per 100,000 by 2018 [16]. Yet, to date, only two studies have investigated the role of *BRCA1* and *BRCA2* mutations in the Lebanese population. These studies reported varied prevalence of pathogenic *BRCA* mutations ranging between 5.6 to 12.5% in BC cases [17, 18]. The reported prevalences of both *BRCA1* and *BRCA2* deleterious mutations were lower than those reported for the Western populations, which suggest the involvement of other genes in the pathogenesis of BC cases [19]. The reported low prevalence does not support the hypothesis that *BRCA1* and *BRCA2* mutations alone are responsible for the majority of the observed Lebanese women with early-onset BC. This finding could well explain the fact that BC is a disease with a high level of genetic heterogeneity and that monogenic and polygenic models of inheritance may exist.

Since the completion of the human genome project, massive leaps have reshaped the field of clinical genomics. The development of Next-generation sequencing (NGS) platforms allowed a more robust, fast and accurate analysis of diseases and syndromes with polygenic nature. NGS platforms including WES are believed to enhance and improve diagnosis and therapy development of many diseases including BC [20–23].

In the presented study, we utilized WES to investigate germline genetic variations in 45 Lebanese cases diagnosed with familial BC and unknown *BRCA1* or *BRCA2* status. We found several rare variants that can potentially explain BC susceptibility in the analyzed cases.

## Methods

### Inclusion criteria

From 2012 to 2015, 45 unrelated patients with inherited BC were selected to undergo DNA testing. They were referred from a wide variety of settings from all over the country, ranging from private physicians’ clinics to major academic medical centers because of hereditary BC. The patients fulfilled a personal history of invasive BC and at least one of the following criteria: A) diagnosis at age ≤ 40 years, B) BC at any age at onset with at least 2 first- and/or second-degree relatives, C) BC < 50 years in a first- or second-degree relative, D) ovarian cancer in at least 2 first- and/or second-degree relatives, E) breast and ovarian cancer in at least 2 first- and/or second-degree relatives, F) both breast and ovarian cancer in a single first- or second-degree relative.

Approval to conduct the study was obtained from the Ethics Committee of Saint-Joseph University-Lebanon. After an informed consent was signed and all ethical requirements were fulfilled, a 10 ml of peripheral blood was isolated from each individual enrolled and the DNA was extracted using the salting out methods [24]. All patients signed the informed consent and agreed to share their variant data.

### Whole exome sequencing

Exon capture and sequencing: Samples were prepared for whole Exome sequencing and enriched according to the manufacturer’s standard protocol. The concentration of each library was determined using Agilent’s QPCR NGS Library Quantification Kit (G4880A). Samples were pooled prior to sequencing with each sample at a final concentration of 10nM. Sequencing was performed on the Illumina HiSeq2000 platform using TruSeq v3 chemistry.

Mapping and alignment: Reads files (FASTQ) were generated from the sequencing platform via the manufacturer’s proprietary software. Reads were aligned to the hg19/b37 reference genome using the Burrows-Wheeler Aligner (BWA) package v0.6.1 [25]. Local realignment of the mapped reads around potential insertion/deletion (Indel) sites was carried out with the Genome Analysis Tool Kit (GATK) v1.6 [26]. Duplicate reads were marked using Picard v1.62. Additional BAM file manipulations were performed with Samtools 0.1.18 [27]. Base quality (Phred scale) scores were recalibrated using GATK’s covariance recalibration. SNP and Indel variants called using the GATK Unified Genotyper for each sample [28]. SNP novelty is determined against dbSNP. A list of 134 genes known to be associated with hereditary BC and other cancers were studied (Additional file 1).

### Variants evaluation

Variants obtained were reported using five categories according to the Human Genome Mutation Database (HGMD Professional) [29]. These categories are listed in Table 1.

**Table 1:** Variants reported in five categories according to the HGMD Professional

Category	Category	Variation reported as
DM	Disease-causing mutations	Pathological mutation
DM?	Disease-causing mutations	Likely pathological mutation
DP	Disease-associated polymorphism	Polymorphism in significant association with a disease/phenotype (*p* < 0.05) that is assumed to be functional
DFP	Disease-associated polymorphism	Polymorphism in significant association with disease (*p* < 0.05) that has evidence of being of direct functional importance
FP	In vitro/laboratory or in vivo functional polymorphism	Polymorphism that affects the structure, function or expression of the gene (or gene product), but with no disease association reported as yet

The first variant category consists of alleles labeled as disease causing mutations (DM) in HGMD Professional. These alleles must be rare: <1% allele frequency in 6,500 exomes from the National Heart, Lung, and Blood Institute (NHLBI) Exome Sequencing Project (“Exome Variant Server” 2015) and the 1,000 Genomes Project Genomes [30].

The *BRCA* gene variants identified were checked for pathogenicity in 4 databases: Breast Cancer Information Core (BIC) [31], Leiden Open Variation Database (LOVD) [32], the Catalogue of Somatic Mutations in Cancer database (COSMIC) [33] and BRCA Exchange website (http://brcaexchange.org) providing data from the ENIGMA consortium [34].

### Variants confirmation

Sanger’s sequencing was utilized to confirm the relevant variants identified by WES and to study the segregation of these variants with the disease phenotype in members of families included in the study. PCR reactions were run in final volumes of 50 μl containing 100 ng DNA, 0.25 mM dNTPs, 100 ng of each primer and 0.02 unit of Taq polymerase (Invitrogen Life Technologies, Carlsbad, CA, USA). PCR was performed in an ABI9700 thermocycler (Applied Biosystems, Foster City, CA) with initial denaturation at 94 °C for 5 min, followed by 35 cycles of 95 °C for 30s, specific annealing temperature for 30s, 72 °C for 30s. Primer sequences are available on request as well as annealing temperatures of each exon. PCR products were purified using the illustraTM GFX PCR DNA and Gel Band Purification Kit (GE Healthcare, Buckinghamshire, UK). Both strands of the products were sequenced using the BigDyeW Terminator v1.1 Cycle Sequencing Kit (Applied Biosystems, Foster City, CA) under standard conditions. The labeled products were subjected to electrophoresis on an ABI3130 and ABI3500 Genetic Analyzer sequencing system (Applied Biosystems, Foster City, CA, USA). Electropherograms were analyzed using Sequence Analysis Software v5.2 (Applied Biosystems, Foster City, CA, USA) and compared to reference sequences using ChromasPro v1.7.6.1 (Technelysium, Queensland, Australia).

## Results

### Patient characteristics and sequencing statistics

**Fig. 1:**
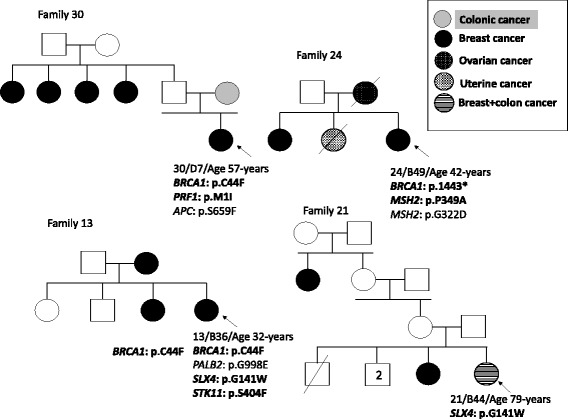
Pedigrees of the families presenting DM mutations. Filled squares (males) and circles (females) indicate the affected individuals. Probands are marked with arrows. DM mutations are bolded

The mean age at diagnosis of BC for the 45 patients was 44 years (range 29–79). Sixteen patients provided us with their histopathological results. Seven BC were estrogen-receptor (ER) and progesterone-receptor (PR) positive, 5 patients had negative ER and PR disease and 2 patients had negative ER and positive PR disease. Two patients had triple negative disease from which one patient (Family 30) carried p.C44F mutation in *BRCA1* (Fig. 1).

We obtained an average of 44 million reads per sample, with a mean coverage of 94% at a mean X coverage of 20X.

### WES analysis

Within this cohort, a total of 126 variants were detected by WES and these are listed in Table 2. In 7 of the 45 patients, not listed in Table 2, no variants in cancer predisposing genes (Additional file 1) were identified.

**Table 2:** Mutations in BC associated genes detected by NGS in a series of 45 Lebanese patients

Family number/Patient code	Genes	Results	HGMD Professional	ExAC allele frequency
1/B19	*XRCC3*	c.C722T p.T241M	DFP Association with melanoma	0.3075
	*XRCC1*	c.G839A p.R280H	DFP Association with increased lung cancer	0.08811
	*CASP8*	c.*429A > G	Not found	Not found
	*BRCA2*	c.C65T p.A22V	Not found	Not found
	*MUTYH*	c.C1258G p.L420V	Not found	Not found
	*SLX4*	c.C1837T p.Q613X	Not found	Not found
2/B21	*BRCA1*	c.A536G p.Y179C	DM Breast and/or ovarian cancer	0.0002718
	*ATM*	c.T2572C p.F858L	DP Association with breast cancer	0.009149
	*ATM*	c.C3161G p.P1054R	DFP Association with breast cancer	0.01692
	*TP53*	c.C215G p.P72R	DFP Association with Lung cancer	0.6600
3/B22	*BRCA2*	c.C5744T p.T1915M	DP Association with breast cancer risk	0.01790
	*ARL11*	c.G446A p.W149X	DP Association with cancer	0.009898
	*TP53*	c.C215G p.P72R	DFP Association with Lung cancer	0.6600
	*ERCC3*	c.C508T p.R170X	Not found	Not found
	*VHL*	c.A631C p.M211L	Not found	0.00004623
	*MRE11A*	c.C1491T p.I497I	Not found	0.0006514
	*PTCH1*	c.G4054A p.V1352I	Not found	Not found
4/B23	*TP53*	c.C215G p.P72R	DFP Association with Lung cancer	0.6600
	*TP53*	c.C245T p.P82L	DM Breast cancer	0.00001657
	*BRCA2*	c.A1114C p.N372H	DFP Association with Breast cancer	0.2779
	*FANCA*	c.G1038C p.W346C	Not found	0.00006621
	*POLE*	c.C3890T p.S1297L	Not found	0.00002580
	*POLD1*	c.T2257C p.Y753H	Not found	Not found
	*GATA2*	c.C1040T p.T347I	Not found	Not found
5/B24	*BRCA2*	c.G8775C p.Q2925H	Not found	0.000008322
	*APC*	c.C6821T p.A2274V	DM Adenomatous polyposis coli	0.0009917
	*EZH2*	c.C349T p.Q117X	Not found	Not found
6/B25	*XRCC3*	c.C722T p.T241M	DFP Association with melanoma	0.3075
	*MLH1*	c.A655G p.I219V	DP Colorectal cancer, non-polyposis	0.2325
	*RAD51D*	c.G494A p.R165Q	Not found	Not found
	*ATM*	c.496 + 4 T > C	Not found	0.00009891
	*PPM1D*	c.G275C p.C92S	Not found	Not found
	*STK11*	c.375-1C > T	DM Colorectal cancer	Not found
7/B26	*XRCC3*	c.C722T p.T241M	DFP Association with melanoma	0.3075
	*MSH2*	c.C1045G p.P349A	DM Renal cell carcinoma	0.00009062
	*MUTYH*	c.C1174A p.L392M	Not found	Not found
	*MUTYH*	c.C1258A p.L420M	DM? Colorectal cancer	Not found
	*ATM*	c.496 + 4 T > C	Not found	0.00009891
	*RB1*	c.C1505T p.T502I	Not found	0.00001098
	*PPM1D*	c.G275C p.C92S	Not found	Not found
8/B27	*XRCC3*	c.C722T p.T241M	DFP Association with melanoma	0.3075
	*BRCA1*	c.A1067G p.Q356R	DP Association with breast and/or ovarian cancer	0.04407
	*XRCC1*	c.C580T p.R194W	DFP Benign breast disease	0.09276
	*CDH1*	c.G1774A p.A592T	DM? Breast cancer	0.003212
35/B28	*BARD1*	c.1071_1091del p.357_364del	Not found	Not found
	*ABCC12*	c.G490T p.G164X	DM Bladder cancer	0.003185
	*MCC*	c.G152T p.G51V	Not found	0.0001346
9/B31	*ATM*	c.T2119C p.S707P	DFP Association with Breast cancer	0.007927
	*FANCA*	c.C4232T p.P1411L	Not found	0.0001318
36/B32	*ATM*	c.C2770T p.R924W	Not found	0.00004942
	*ALK*	c.T4211C p.L1404P	Not found	0.00008370
10/B33	*BRIP1*	c.A3571G p.I1191V	Not found	0.00004967
	*NSD1*	c.2224_2243del p.P742fs	Not found	Not found
	*FANCG*	c.G1298C p.R433P	Not found	0.00004118
	*FLCN*	c.T1387C p.Y463H	Not found	0.00003298
	*PTCH1*	c.A3749G p.Y1250C	Not found	Not found
11/B34	*XRCC4*	c.T401C p.I134T	DP Association with Lung cancer	0.02505
	*RAD51C*	c.G376A p.A126T	FP Reduced activity	0.003529
12/B35	*ARL11*	c.G571A p.G191R	Not found	0.00002188
	*Rad50*	c.A280C p.I94L	DM? Breast and/or ovarian cancer	0.003473
	*POLE*	c.G2276A p.R759H	Not found	0.00001647
13/B36	*BRCA1*	c.G131T p.C44F	DM Breast and/or ovarian cancer	Not found
	*SLX4*	c.G421T p.G141W	DM Breast and/or ovarian cancer	0.0008237
	*STK11*	c.C1211T p.S404F	DM Peutz-Jeghers syndrome	0.0009281
	*PALB2*	c.G2993A p.G998E	DP Breast cancer, increased risk-	0.01579
	*BRCA2*	c.C1151T p.S384F	DM? Breast cancer	0.0006789
	*DICER1*	c.A5276G p.K1759R	Not found	0.00004942
	*CEBPA*	c.T122C p.I41T	Not found	Not found
	*RECQL4*	c.G3314A p.G1105D	Not found	0.005430
14/B37	*RAD50*	c.G379A p.V127I	Not found	0.001653
	*CASP8*	c.A1117G p.I373V	Not found	Not found
	*RECQL4*	c.C3184T p.R1062W	Not found	0.0003129
	*WRN*	c.G4129A p.G1377S	Not found	0.00002483
15/B38	*BARD1*	c.C119T p.A40V	Not found	0.00004775
	*PTCH1*	c.169_170delCT p.57_57del	Not found	0.000008913
	*PTCH1*	c.A3749G p.Y1250C	Not found	Not found
	*PTCH1*	c.C4126T p.R1376W	Not found	Not found
	*ERCC5*	c.A1904G p.H635R	Not found	Not found
	*DICER1*	c.C3811T p.L1271F	Not found	Not found
16/B39	*CDKN2A*	c.G442A p.A148T	DP Association with melanoma	0.02278
	*RAD51D*	c.A758G p.E253G	Not found	0.01144
	*ERCC6*	c.C2800A p.P934T	DM Cockayne syndrome	Not found
17/B40	*MSH2*	c.A1787G p.N596S	DM Colorectal cancer, non-polyposis	0.0002558
	*ATM*	c.A1982C p.D661A	Not found	Not found
	*PMS2*	c.G1688T p.R563L	DM? Colorectal cancer, non-polyposis	0.005813
	*GPC3*	c.78_79insCCG p.P27delinsPP	Not found	Not found
18/B41	*BRCA2*	c.658_659delGT p.V220I*	DM Breast and/or ovarian cancer	0.00006119
	*SLX4*	c.G3337C p.G1113R	Not found	0.000008237
	*SMARCA4*	c.C1098G p.I366M	Not found	0.00002715
	*EPHX1*	c.G1040C p.R347T	Not found	0.00003296
19/B42	*wwoxtv2*	c.A544G p.K182E	DM? cancer	Not found
	*ATM*	c.A5558T p.D1853V	DP Association with breast cancer, contralateral	0.005186
	*RET*	c.C2508T p.S836S	DP Association with thyroid cancer	0.04666
	*BRCA1*	c.5090_5093delGTTA p.L1697fs	Not found	Not found
20/B43	*PALB2*	c.G2014C p.E672Q	DM? Breast cancer? (common variant)	0.02239
	*PALB2*	c.G2993A p.G998E	DP Breast cancer (common variant)	0.01579
	*RAD51C*	c.G376A p.A126T	FP Reduced activity	0.003529
	*Tp53*	c.673-36G > C	DFP Breast cancer	Not found
21/B44	*SLX4*	c.G421T p.G141W	DM Breast and/or ovarian cancer	0.0008237
	*SLX4*	c.C1919A p.T640N	Not found	Not found
	*FANCM*	c.A5224G p.I1742V	Not found	0.008398
	*POLD1*	c.G2793C p.K931N	Not found	Not found
22/B45	*ATM*	c.A5071C p.S1691R	DM Ataxia telangiectasia	0.002019
	*BRIP1*	c.G2220T p.Q740H	DM? Breast and/or ovarian cancer	0.0004614
	*RET*	c.C2508T p.S836S	DP Association with thyroid cancer	0.04666
	*FANCA*	c.A796G p.T266A	DP Associated with breast cancer	0.5166
23/B46	*BARD1*	c.1071_1091del p.357_364del	Not found	Not found
	*FANCA*	c.C3412G p.L1138V	Not found	0.001533
	*MRE11A*	c.A1728T p.R576R	Not found	0.000008238
	*SLX4*	c.C1186G p.L396V	Not found	Not found
37/B47	*SLX4*	c.A5501G p.N1834S	Not found	0.005542
	*ERCC4*	c.G1633C p.G545R	Not found	0.000008243
38/B48	*SDHC*	c.C31T p.R11C	Not found	0.000008252
	*FANCD2*	c.A1348G p.I450V	Not found	0.0003871
	*FANCF*	c.C959T p.P320L	Not found	0.01264
	*TSC2*	c.A2834G p.K945R	Not found	Not found
	*DIS3L2*	c.1651_1652insGGG p.A551delinsGA	Not found	Not found
	*GNAS*	c.C1046T p.P349L	Not found	Not found
24/B49	*BRCA1*	c.C4327T p.R1443*	DM Breast cancer	Not found
	*MSH2*	c.C1045G p.P349A	DM Renal cell carcinoma	0.00009062
	*MSH2*	c.G965A p.G322D	DM? Colorectal cancer, non-polyposis	0.01411
	*BARD1*	c.G253T p.V85L	Not found	0.001068
	*NBN*	c.G340T p.V114F	Not found	Not found
	*RET*	c.C2249G p.A750G	Not found	0.000008238
	*XRCC3*	c.C260T p.P87L	Not found	0.00006286
25/B50	*POLH*	c.A2074G p.T692A	DM Xeroderma pigmentosum	0.0001824
	*Tp53*	c.673-36G > C	DFP Breast cancer	Not found
	*CTNNB1*	c.A2315G p.N772S	Not found	0.00003355
	*POLD1*	c.C519G p.S173R	Not found	0.009212
26/D1	*ARL11*	c.G446A p.W149X	DP Association with cancer	0.009898
	*MSH2*	c.T1182G p.F394L	Not found	0.00001648
27/D4	*CHEK2*	c.T470C p.I157T	DFP Li-Fraumeni syndrome	Not found
28/D5	*CDH1*	c.G2387A p.R796Q	Not found	0.00003300
29/D6	*BUB1B*	c.A1535G p.E512G	Not found	0.000008239
30/D7	*APC*	c.C2876T p.S959F	Not found	Not found
	*BRCA1*	c.G131T p.C44F	DM Breast and/or ovarian cancer	Not found
	*PRF1*	c.G3A p.M1I	DM Haemophagocytic lymphohistiocytosis, familial	Not found
31/D8	*TP53*	c.G469A p.V157I	DM Sarcoma, adult-onset	0.00005776
32/III_4	*CDH1*	c.G3A p.M1I	DM Gastric cancer	Not found
	*BRCA2*	c.C4061T p.T1354M	DM Breast cancer	0.000008328
	*BRCA2*	c.G4258T p.D1420Y	DM? Breast and/or ovarian cancer	0.006796
33/D12	*CDH1*	c.A160G p.R54G	Not found	0.00005916
34/D13	*BRCA2*	c.G223C p.A75P	DM? Breast cancer	0.0001650

DM disease-causing mutation, DM? likely disease-causing mutation, DP disease-associated polymorphism, FP *in vitro* or *in vivo* functional polymorphism, DFP disease-associated polymorphism with additional functional evidence

We were able to detect 19 HGMD DM variations of which 9 are specifically associated with breast cancer (Table 2). The distribution of the remaining variants in the HGMD categories was: 11 DM?, 11 DP, 1 FP, and 9 DFP. In addition, 75 novel variations were detected in this study (Table 2).

Six *BRCA1* and *BRCA2* DM mutations were detected in 5 and 2 patients, respectively in a total prevalence of 15.5% (Table 2).

Nine truncating mutations were detected in 9 different patients (Table 2). Three of these mutations were DM in HGMD: The first woman carried p.R1443* in *BRCA1*, the second one carried p.V220I* in *BRCA2* and the third one carried p.G164X in *ABCC12* (Table 2). The six remaining truncating mutations were not found in HGMD: p.Q613X in *SLX4*, p.R170X in *ERCC3*, p.Q117X in *EZH2*, p.P742fs in *NSD1*, p.357_364del in *BARD1* and p.L1697fs in *BRCA1* (Table 2).

Three DM mutations were found, each one, in 2 different patients: p.C44F in *BRCA1* (Families 13 and 30), p.P349A in *MSH2* (Families 7 and 24) and p.G141W in *SLX4* (Fig. 1 and Table 2).

**Fig. 2:**
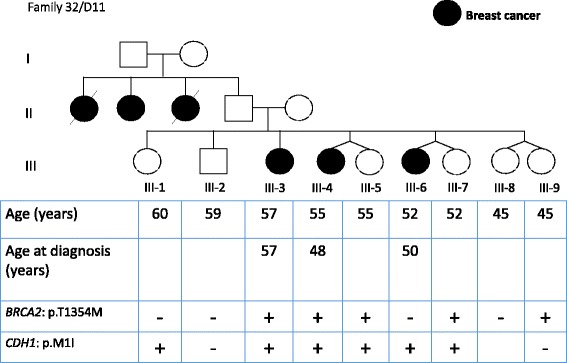
Pedigree of one family segregated for *BRCA2* p.T1354M and *CDH1* p.M1I variants. Filled squares (males) and circles (females) indicate the affected individuals. + sign indicates the presence of the variant and – sign the absence of the variant in tested individuals

In some families where different variants were found, in order to consider, which variant is pathogenic, we analyzed the co-segregation of the variations found with the cancer phenotype within 3 families 12, 13, and 32 (Figs. 1 and 2).

Two members of family 12 were diagnosed with BC, their mother and maternal uncle were diagnosed with primary lung cancer and bone cancer, respectively. The nonsmoking mother was affected at the age of 63 but the age of the maternal uncle at diagnostic was not accessible. WES, in proband 12/B35 diagnosed with BC at the age of 42, identified 2 variants including one DM? p.I94L in *RAD50*, according to HGMD Professional database, and one novel variation p.G191R in *ARL11* (data not shown). Prediction tool Polyphen2 indicated that both changes are benign and SIFT prediction tool indicated that p.I94L in *RAD50* is tolerated and that p.G191R in *ARL11* is damaging. Only p.I94L in *RAD50* segregated in the affected sisters, diagnosed with BC at the age of 48, but it was also found in their third youngest 51 years old unaffected sister.

Three members of Family 13 were diagnosed with BC. WES identified 3 DM according to HGMD Professional database, including p.C44F in *BRCA1*, p.G141W in *SLX4* and p.S404F in *STK11* (Table 2). Leiden Open Variation Database indicated that p.C44F in *BRCA1* affects protein function and it segregated with the disease (Table 3) (Fig. 1).

**Table 3:** *BRCA* variations found and their evaluations in *BRCA* databases

Gene	Variation	BIC database Clinically Importance/ Clinical Classification	COSMIC	Leiden Open Variation Database (LOVD)	BRCA Exchange
*BRCA1*	c.G131T p.C44F	unknown/ pending	Not found	Affects function	Not found
	c.A536G p.Y179C	unknown/ pending	Not found	Does not affect function	Benign
	c.C4327T p.R1443*	yes/ class 5	Neutral	Affects function	Not found
	c.A1067G p.Q356R	unknown/ pending	Pathogenic	Does not affect function	Benign
	c.5090_5093delGTTA p.L1697fs	Not found	Not found	Not found	Not found
*BRCA2*	c.C65T p.A22V	unknown/pending	Not found	Effect unknown	Not found
	c.G223C p.A75P	unknown/ pending	Not found	Does not affect function	Benign
	c.658_659delGT p.V220I*	yes/ class 5	Not found	Affects function	Not found
	c.C4061T p.T1354M	unknown/ pending	Neutral	Does not affect function	Benign
	c.G4258T p.D1420Y	no/ pending	Neutral	Does not affect function	Benign
	c.C5744T p.T1915M	no/ class 1	Neutral	Does not affect function	Not found
	c.G8775C p.Q2925H	unknown/ pending	Not found	Effect unknown	Not found
	c.A1114C p.N372H	no/ class 1	Neutral	Not found	Benign
	c.C1151T p.S384F	no/ pending	Not found	Not found	Benign

Descriptions of the classes in the BIC database:

Class 1: Not pathogenic/low clinical significance: There is significant evidence against this variant being a dominant high-risk pathogenic mutation

Class 5: Pathogenic: There is significant evidence to suggest that this variant is a dominant high-risk pathogenic mutation

Six members of family 32 were diagnosed with BC (Fig. 2). Members III-3, III-4 and III-6 were diagnosed with BC at the age of 56, 48 and 50, respectively. WES in proband III-4 identified 2 relevant variants including p.M1I in *CDH1* and p.T1354M in *BRCA2*. Prediction tool SIFT indicated that both changes are damaging and are DM according to HGMD Professional database (Table 2 and Fig. 2). The analysis of this family showed that these variations were carried by affected and siblings that are not affected to date (Fig. 2). However, they were advised to join our screening program.

**Fig. 3:**
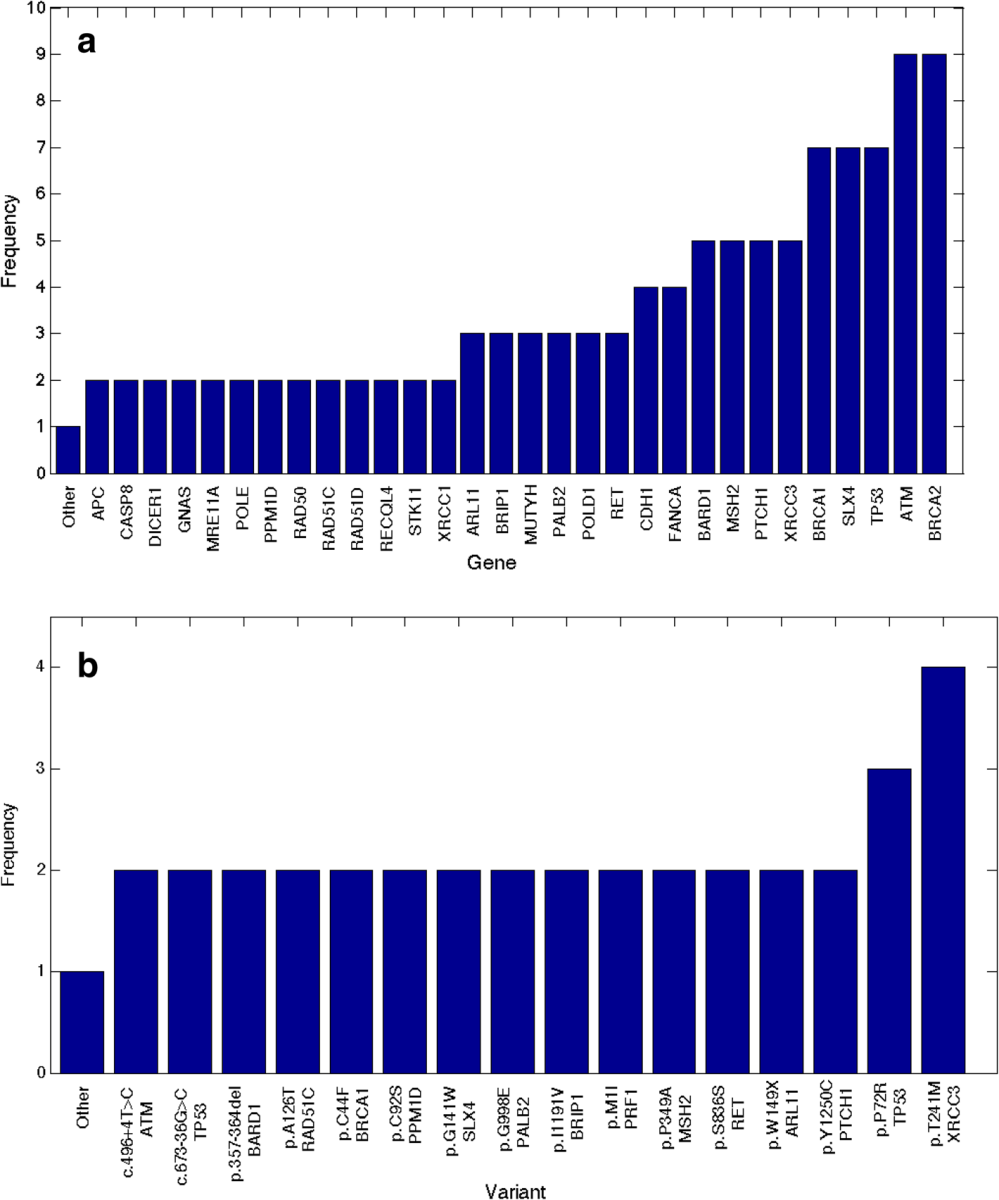
Frequencies of all variants in breast cancer predisposing genes from our 45 patients’ cohort (**a**) and the details of the most frequent variants shown in (**b**)

We have noted that the most frequently altered genes involved in our familial cases are DNA repair genes (Fig. 3a) and that some variants were recurrent in our cohort: p.W149X in *ARL11*, p.S836S in *RET*, p.A126T in *RAD51C*, p.T241M in *XRCC3*, p.G998E in *PALB2* and c.673-36G > C in *TP53* (Table 2 and Fig. 3b). In four cases, like the 4 families shown in Fig. 1, individuals appear to co-inherit multiple cancer causing or predisposing gene mutations. Unlike, the old strategy where one stops the investigation once a pathogenic mutation was identified, NGS gives us the capability of collating all known mutations/variants in a sample, which may permit a more comprehensive understanding of the polygenic landscape model of cancer. An important question to be answered is: Does an individual in Family 13 harboring all three DM mutation have different penetrance, genotype to phenotype correlation, type or age of onset of cancer than a sibling with only one DM variant? This critical question can only be answered when we start to combine all germline variant data of cancer patients and their comprehensive phenotypes from around the world in well-curated databases.

## Discussion

We identified, in 45 patients with familial BC, 19 pathogenic mutations that are DM mutations according to the HGMD Professional database (Table 2). These 19 mutations were found in 13 different genes including *ABCC12*, *APC*, *ATM*, *BRCA1*, *BRCA2*, *CDH1, ERCC6*, *MSH2*, *POLH*, *PRF1, SLX4*, *STK11,* and *TP53*. Six mutations were found in *BRCA1* and *BRCA2* presenting a lower prevalence (15.5%) of deleterious *BRCA* mutations compared to the published literature [21–23].

In the Lebanese population, p.C44F mutation in the *BRCA1* gene was found twice in this study and 5 times in previous studies [17, 18] in a total of 7 from 367 cases studied (1.9%). In fact, 2 of 9 patients carried a deleterious BRCA mutation in a cohort of 72 patients and 3 of 14 patients carried a deleterious BRCA mutation in a cohort of 250 patients. Our findings suggest it is the most recurrent mutation in the Lebanese population.

In families 23 and 35, we identified the truncating mutation p.357_364del in *BARD1* (Table 2). A previous study, on this variation, showed the absence of co-segregation with the disease and it was considered as neutral polymorphisms [35]. We have observed this variant in our population and breast cancer patients and it is recommended that a more thorough and functional examination of this variant be conducted in the future.

In families 12, 13 and 32, we identified 7 variants in *ARL11*, *BRCA1*, *BRCA2, CDH1, RAD50, SLX4*, and *STK11*. The association of which variation towards increasing predisposition to BC remains unknown. Therefore, we analyzed the segregation of these variations and BC within the families. In family 13, only p.C44F in *BRCA1* segregated with BC in the family. In family 12, p.I94L in *RAD50* (a DM? mutation) was found in affected and healthy sisters and could therefore not lead to a conclusion regarding predisposition to BC. In family 32, p.M1I in *CDH1* and p.T1354M in *BRCA2* are implicated in gastric cancer and BC respectively and knowing that the family presented with only BC, two hypothesis can be formulated. First, III-6 can be considered as phenocopy and second healthy, till now, sisters III-5, III-7 and III-9 are at high risk (Fig. 2). In fact, in high-risk families, women testing negative for the familial *BRCA* mutation have an increased risk of BC and should be considered for continued surveillance [36]. Interestingly, two members of this family, III-4 and III-6 presented with invasive lobular breast cancer (Fig. 2). The association between *CDH1* gene mutation and lobular cancer has been well established previously [37], and it is not unrealistic to suggest that this *CDH1* variant may be the cause of lobular breast cancer in this family.

The pathogenic status of the majority of novel substitutions found and the 6 variations considered as DM? according to HGMD professional, remains problematic (Table 2). In fact, HGMD professional reports DM? as likely pathological mutation reported to be disease causing in the corresponding report, but the author has indicated that there may be some degree of doubt, or subsequent evidence has come to light in the literature, calling the deleterious nature of the variant into question [29]. Further studies are needed to define the pathogenic status of the novel substitutions and the DM? variations that have been found in our cohort of patients with BC. These future studies have to be analyzed in a larger number of affected families and control population samples.

NGS and traditional sequencing methods are not proficient in detecting *BRCA* genomic rearrangements including large deletions or duplications. Deletion and duplication genomic rearrangements vary significantly among countries and within ethnic groups [38]. We admit, therefore, that our reported *BRCA* mutation prevalence is underestimated.

Among the DM mutations found, several were associated with syndromes (Peutz-Jeghers), different cancer types (renal cell carcinoma, gastric cancer) and with diseases (Xeroderma pigmentosa, ataxia telangiectasia) (Table 2). Clinically, none of the symptoms found in these diseases were manifested in the different studied families except for family 24. In this family, proband 24/B49 carried the mutation p.R1443* in *BRCA1* and two *MSH2* variants (Fig. 1). Her mother had ovarian cancer and her sister uterine cancer, both are deceased and could not consequently be tested for these variants. *MSH2* mutation is reported in families with endometrial cancer (Lynch syndrome) and breast cancer from Kuwait [39].

This is the first application of NGS on BC in Lebanon. In this study, we showed that the prevalence of deleterious *BRCA* mutations (15.5%) is lower than expected [17, 18] and that the overall mutation prevalence is equal to 40%, justifying the urgent need for the adoption of high-throughput NGS technologies to identify genes responsible for familial BC in the Lebanese population. Indeed, additional to *BRCA* mutations, highly penetrant mutations in genes associated with various hereditary cancer syndromes, such as *CDH1*, *TP53*, *MSH2, ATM* and *POLH* were found in the Lebanese population. Finally, we cannot rule out that some of these families shift a putative explanation towards a polygenic model where moderate and low penetrance alleles, acting together, may play a predominant role [20, 40, 41]. Our findings support the eligibility of performing genetic testing by massively parallel sequencing on Lebanese familial BC cases. Moreover, we would like to use this technology for tumor genome sequencing, in order to identify somatic alterations, which would be a valuable guidance towards individualized cancer therapy of Lebanese patients with BC. However, it is worthy of note that our study reports a small number of variants that are clinically actionable. Given the high rate of novel variants identified in *BRCA1/2* and other breast cancer-associated genes, the clinical usefulness of the data is currently limited. Unless larger and rigorous studies are committed in this area of the world to correctly classify variants identified here or in other studies, the diagnosis and treatment of breast cancer will remain suboptimal.

## Conclusion

This is the first study that utilized NGS technology to study genetic variants in 45 patients with familial breast cancer from Lebanon. Our deleterious mutation prevalence was 40% with only 15.5% accounted for by the *BRCA1* and *BRCA2* genes. This data should encourage a different strategy for familial breast cancer genetic screening in Lebanon, one that is based on WES rather than the initial screening of *BRCA1/2* genes. We report here novel and rare variants in breast cancer predisposing genes, which will be valuable to researchers and clinicians around the world for variants’ classification and patients’ care in general.

## Additional file

Additional file 1: Cancer genes explored in this study. (DOCX 11 kb)

## Abbreviations

BC: Breast cancer
BIC: Breast cancer information core
BWA: Burrows-wheeler aligner
COSMIC: Catalog of somatic mutations in cancer database
DFP: Disease-associated polymorphism
DM: Disease-causing mutations
DM?: Disease-causing mutations
DP: Disease-associated polymorphism
FP: In vitro/laboratory or in vivo functional polymorphism
GATK: Genome analysis tool kit
HGMD Professional: Human genome mutation database
LOVD: Leiden open variation database
NGS: Next generation sequencing
NHLBI: National heart, lung, and blood institute
WES: Whole exome sequencing.
