# Supplementary material for: Next-generation sequencing in familial breast cancer patients from Lebanon
Source: BMC Med Genomics. 2017 Feb 15;10:8. doi: 10.1186/s12920-017-0244-7 (PMC5312584; doi:10.1186/s12920-017-0244-7)
Supplement: Additional file 1: — Cancer genes explored in this study. (DOCX 11 kb) [file 12920_2017_244_MOESM1_ESM.docx]

**Additional file 1**

**Cancer genes explored in this study**

*ABCC12, AIP, ALK, APC, AR, ARL11, ARL17A, ATM, AXIN2, BAP1, BARD1, BLM, BMPR1A, BRCA1, BRCA2, BRIP1, BUB1B, CASP8, CAV1, CDC73, CDH1, CDH3, CDK4, CDKN1C, CDKN2A, CEBPA, CEP57, CHEK2, CTNNB1, CYLD, DDB2, DIRAS3, DICER1, DIS3L2, EGFR, EPHX1, EPCAM, ERBB2, ERCC2, ERCC3, ERCC4, ERCC5, ERCC6, EXT1, EXT2, EZH2, FANCA, FANCB, FANCC, FANCD2, FANCE, FANCF, FANCG, FANCI, FANCL, FANCM, FGFR3, FH, FLCN, GATA2, GNAS, GPC3, HNF1A, HRAS, HSD17B2, IFI27, KIT, MAX, MCC, MEN1, MET, MITF, MLH1, MRE11A, MSH2, MSH6, MUTYH, NBN, NF1, NF2, NSD1, PALB2, PHOX2B, PMS1, PMS2, POLD1, POLE, POLH, PPM1D, PRF1, PRKAR1A, PTCH1, PTEN, RAD50, RAD51, RAD51C, RAD51D, RB1, RECQL, RECQL4, RET, RHBDF2, RUNX1, SBDS, SDHA, SDHAF2, SDHB, SDHC, SDHD, SLX4, SMAD4, SMARCB1, SMARCA4, STK11, SUFU, TGFB1, TMEM127, TOP2A, TOPBP1, TP53, TP73, TPD52, TSC1, TSC2, VHL, WRN, WWOXTV2, WT1, XPA, XPC, XRCC1, XRCC2, XRCC3, XRCC4.*
